# P-441. Epidemiology of Pediatric Mycoplasma pneumoniae (MP) Infections at Two Pediatric Hospitals in Los Angeles County (LAC), January 1, 2017 to January 31, 2025

**DOI:** 10.1093/ofid/ofaf695.656

**Published:** 2026-01-11

**Authors:** Heidi Ransohoff, Huan V Dong, Natalie Quanquin, Elizabeth Traub, Sanchi Malhotra, Ishminder Kaur, Annabelle de St Maurice

**Affiliations:** Los Angeles Department of Public Health, Los Angeles, California; UCLA, Los Angeles, CA; Miller Children's and Women's Hospital Long Beach, Long Beach, California; Los Angeles County Department of Public Health, Los Angeles, CA; University of California, Los Angeles, Los Angeles, California; Mattel Children's Hospital at UCLA, Los Angeles, CA; University of California Los Angeles, Los Angeles, California

## Abstract

**Background:**

In May 2024, LAC Department of Public Health (DPH) received reports about increased pediatric MP infections. In October 2024, the U.S. Centers for Disease Control and Prevention (CDC) reported a national increase in MP infections and hospitalizations among young children. We describe pediatric MP infections from 2017 to 2025 in LAC and compare case characteristics between pre- and post-pandemic periods.

**Figure:**
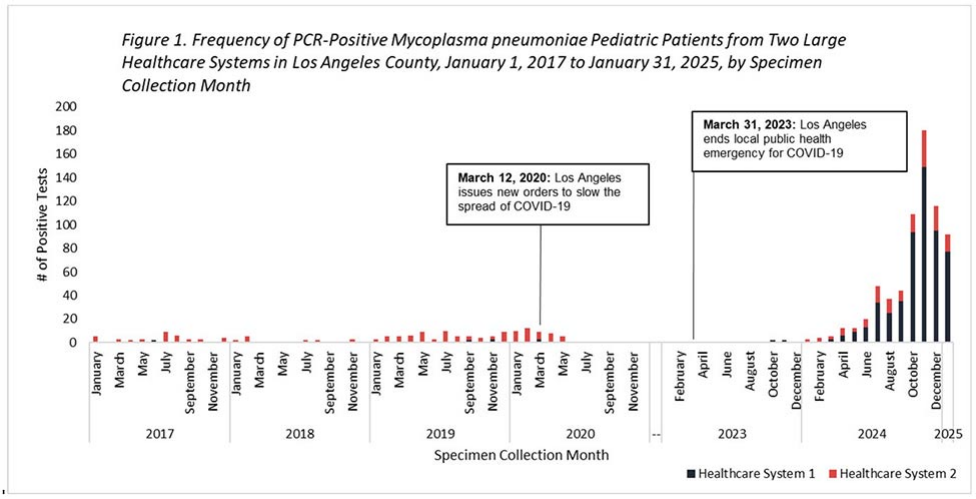

**Figure:**
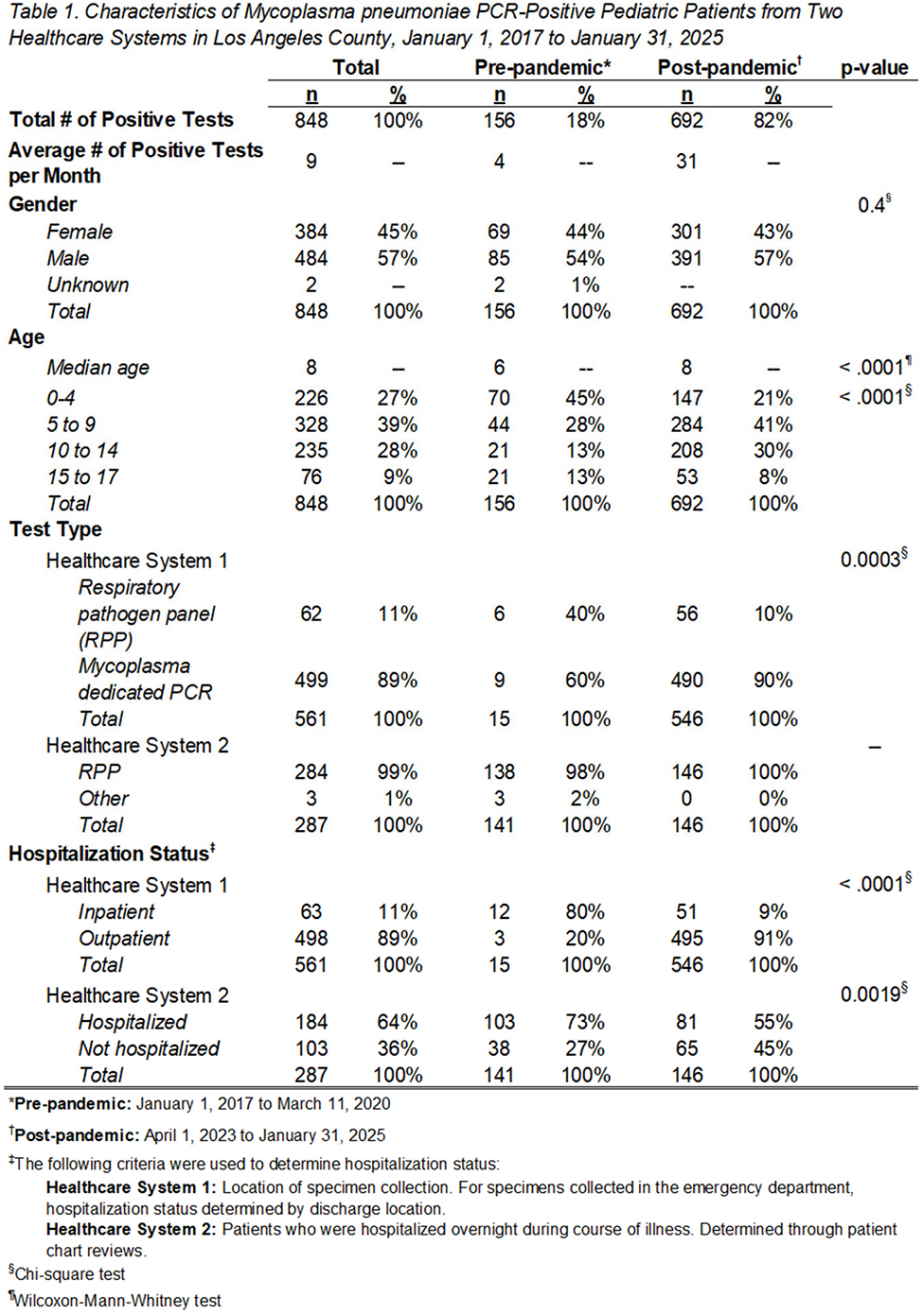

**Methods:**

LAC DPH conducted a retrospective cohort study of patients < 18 years seen at two LAC healthcare systems (HS1 and HS2) who tested MP positive by respiratory pathogen panel (RPP) or single target molecular test between January 1, 2017 and January 31, 2025. Positive patients were identified through lab data; HS2 also used electronic health records. Pre- and post-pandemic were defined as before March 12, 2020 and after March 31, 2023, respectively. χ2and Wilcoxon-Mann-Whitney tests were used to assess relationships between characteristics and pre- and post-pandemic periods. Data analysis was performed in SAS 9.4.

**Results:**

Of 865 positive patients, 561 were reported from HS1; 287 were reported form HS2. 156 infections occurred pre-pandemic, 22 during the pandemic, and 687 post-pandemic (Figure 1). On average, there were 4 positive patients reported per month pre-pandemic and 31 positive patients reported per month post-pandemic. HS1 and HS2 reported fewer inpatient positive patients post-pandemic compared to pre-pandemic (p < .05; Table 1). HS1 detected 11% of their tests via RPP; HS2 detected 99% of their tests via RPP.

Overall, 484 (56%) patients were male and 384 (44%) were female. Pre- and post-pandemic, the median age was 6 and 8 years old, respectively (p < .0001).

**Conclusion:**

The number of MP positive patients and proportion of outpatient cases greatly increased post-pandemic, which may be due to increased testing of mild cases. Data on changes in age distribution of cases post-pandemic are varied; in this study the median age of patients increased after the pandemic, which may be due to a larger susceptible population post-pandemic. The proportion of cases identified using an RPP versus a dedicated PCR varied between HS1 and HS2, which may affect sensitivity. Further studies should be conducted to better understand MP epidemiology post-pandemic.

**Disclosures:**

All Authors: No reported disclosures

